# Effective Non-Invasive Delivery of Epigenetic Drugs Using Functionalized Accessory Unit Conjugates

**DOI:** 10.3390/pharmaceutics18010115

**Published:** 2026-01-15

**Authors:** Toshihiko Tashima

**Affiliations:** Tashima Laboratories of Arts and Sciences, 1239-5 Toriyama-cho, Kohoku-ku, Yokohama 222-0035, Kanagawa, Japan; tashima_lab@yahoo.co.jp

**Keywords:** epigenetics, epigenetic drug delivery, nanoparticle-mediated delivery, antibody–drug conjugate, receptor-mediated endocytosis, carrier-mediated transport, cancer therapy

## Abstract

Epigenetics involves heritable changes in gene expression—such as DNA methylation (5-methylcytosine; 5mC), histone modifications, and regulation by non-coding RNAs at the mRNA translation level—without altering the underlying DNA sequence. As targeting these mechanisms enables intervention at the root cause of disease rather than the symptoms alone, epigenetics has become a rapidly advancing field in pharmaceutical sciences. Various epigenetic modulators, including histone deacetylase (HDAC) inhibitors, histone acetyltransferase (HAT) inhibitors, DNA methyltransferase (DNMT) inhibitors, and microRNAs (miRNAs), have been developed, and some have already been approved for cancer therapy. However, these agents often face significant challenges such as poor membrane permeability, enzymatic instability, and suboptimal biodistribution. Incorporating functionalized accessory units—serving as vectors (e.g., transporter recognition units, cell-penetrating peptides, tumor-homing peptides, monoclonal antibodies) or as carriers (e.g., monoclonal antibodies, nanoparticles)—into epigenetic modulators may help overcome these delivery barriers. In this narrative review, I discuss the potential and advantages of effective non-invasive delivery of epigenetic drugs using such functionalized accessory unit conjugates.

## 1. Introduction

The complete sequencing of the human genome [1] has revealed that protein-coding genes occupy only approximately 1.5% of the total genome. While humans possess roughly 23,000 genes [2], the fruit fly (*Drosophila melanogaster*) has about 13,500 genes [3]. Therefore, organismal complexity does not necessarily correlate with the number of genes. However, the human genome contains a significantly larger proportion of non-coding regions than that of any other species, suggesting that epigenetic regulation [4] plays a crucial role in biological development, differentiation, and morphology. Moreover, epigenetic alterations are closely associated with the etiology and pathology of various diseases, including cancer, metabolic disorders, and neurodegenerative diseases [5]. Remarkably, induced pluripotent stem cells (iPSCs) can be generated from differentiated somatic cells through epigenetic reprogramming mediated by the introduction of four transcription factors—Oct3/4 (octamer-binding transcription factor 3/4), Sox2 (SRY-box transcription factor 2), c-Myc (MYC proto-oncogene, bHLH transcription factor), and Klf4 (Kruppel like factor 4)—known as the Yamanaka factors that can completely change a regular adult cell into a pluripotent stem cell [6]. However, iPSCs often retain epigenetic memory, mainly through mechanisms such as DNA methylation [7]. Accordingly, epigenetic modulators are regarded as promising pharmaceutical agents for treating diseases at their root cause rather than merely alleviating symptoms [8].

**Figure 1:**
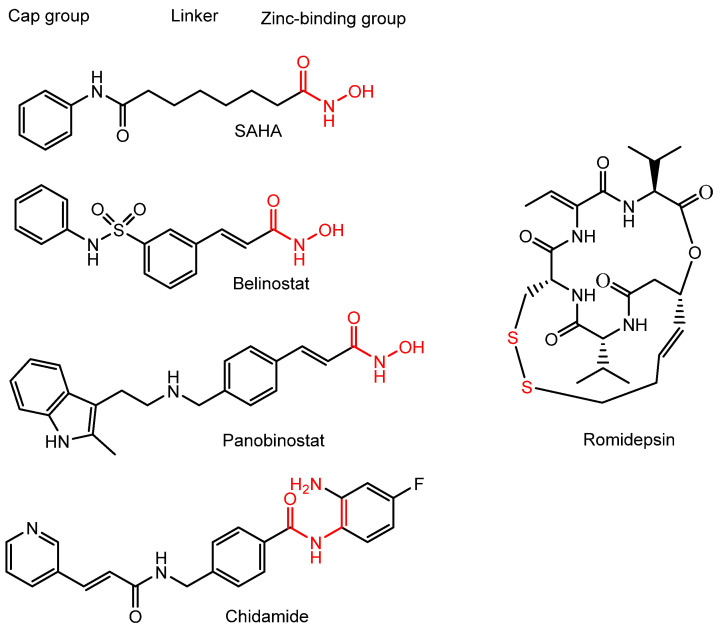
Chemical structures of clinically approved histone deacetylase (HDAC) inhibitors, including SAHA (vorinostat), belinostat, panobinostat, chidamide, and romidepsin. From a drug design perspective, HDAC inhibitors are structurally composed of a cap group and a zinc-binding group (ZBG) connected via an appropriate linker. Representative zinc-binding groups (shown in red) include hydroxamic acid, benzamide, and thiol moieties. Romidepsin is considered a prodrug, as it undergoes intracellular reduction to release the active thiol-containing inhibitor.

Phenomenologically, DNA methylation (5-methylcytosine; 5mC) and histone modifications represent two major types of epigenetic marks. Additionally, non-coding RNAs such as microRNAs (miRNAs) and small interfering RNAs (siRNAs) are known to regulate mRNA translation. To date, numerous epigenetics-targeted drugs have been developed [9,10]. Among them, histone deacetylase (HDAC) inhibitors—including suberoylanilide hydroxamic acid (SAHA; vorinostat), belinostat, panobinostat, and chidamide—are clinically approved anticancer agents (Figure 1). These inhibitors typically contain either a hydroxamic acid group or a benzamide group as the zinc-binding moiety, which contributes to their activity but also results in poor membrane permeability and off-target effects. Chidamide, approved by the China Food and Drug Administration in 2014 for the treatment of relapsed or refractory peripheral T-cell lymphoma, features a benzamide zinc-binding group (Figure 1).

**Figure 2:**
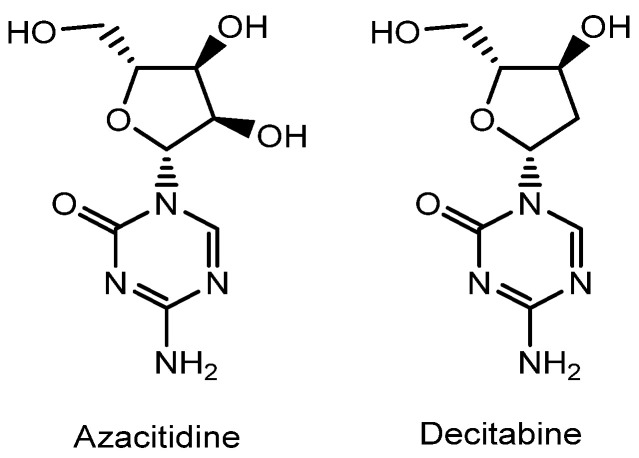
Chemical structures of clinically approved epigenetic modulators other than histone deacetylase (HDAC) inhibitors, small interfering RNAs (siRNAs), and antisense oligonucleotides. Azacitidine and decitabine act as DNA methyltransferase (DNMT) inhibitors.

Membrane impermeability can potentially be improved by conjugating these drugs with vectors such as cell-penetrating peptides (CPPs), tumor-homing peptides (THPs), monoclonal antibodies, or nanoparticles. Conversely, off-target effects may be mitigated by using carriers such as nanoparticles or antibodies that provide steric hindrance, preventing undesired interactions with non-target molecules. In addition to HDACs, several other key enzymes participate in epigenetic regulation, including histone acetyltransferases (HATs), histone lysine methyltransferases (HKMTs), histone lysine demethylases, DNA methyltransferases (DNMTs), and bromodomain and extra-terminal (BET) proteins [11]. DNMT inhibitors, such as azacitidine and decitabine, are hydrophilic and therefore exhibit limited membrane permeability [8] (Figure 2). Furthermore, RNAs are inherently susceptible to enzymatic degradation by ribonucleases (RNases). To address this, encapsulation of miRNAs, siRNAs, and antisense oligonucleotides within nanoparticles can provide significant protection and enhance delivery efficiency. In this narrative review, I introduce the possibilities and strategies for effective, non-invasive epigenetic drug delivery by employing functionalized accessory units—including vectors such as transporter recognition units, CPPs, THPs, and monoclonal antibodies, as well as carriers such as monoclonal antibodies and nanoparticles.

## 2. Overview of Delivery Strategies

### 2.1. Conjugates with Transporter Recognition Units

The use of conjugates containing transporter recognition units enables efficient drug delivery. The blood–brain barrier (BBB) prevents most circulating drugs from entering the brain, posing a major challenge in the discovery and development of central nervous system (CNS) therapeutics [12]. The BBB consists primarily of: (a) physical barriers, including tight junctions between capillary endothelial cells, the hydrophobic lipid bilayer of cell membranes, and the perivascular structures formed by pericytes and astrocytes; and (b) biological barriers, such as active efflux by transporters, notably multiple drug resistance protein 1 (MDR1, P-glycoprotein) [13]. Compounds are generally classified by molecular weight (MW) as low-molecular-weight compounds (MW < ~500), middle-molecular-weight compounds (MW ~500–3000), and high-molecular-weight compounds (MW > ~3000) [14]. From a physical standpoint, filtration depends primarily on molecular size, a principle that is also applied in gel filtration chromatography. The behavior of acids and bases is explained by the Arrhenius, Brønsted–Lowry, and Lewis theories. Consequently, the pKa value represents the acidity constant of a given molecule. Positively charged species electrostatically interact with negatively charged species. Hydrophobic materials exhibit low affinity toward hydrophilic materials. Hydrophobic interactions are non-covalent forces that play crucial roles in molecular binding, membrane passive permeation, and aggregation. At the BBB, however, hydrophobic low-molecular-weight compounds are frequently expelled by efflux transporters such as MDR1, while hydrophilic compounds cannot readily cross the lipid bilayer via passive diffusion due to electrostatic repulsion and lack of membrane affinity. For instance, HDAC inhibitors containing hydrophilic zinc-binding groups (e.g., hydroxamic acid or benzamide) exhibit poor passive transcellular permeability. Solute carrier (SLC) transporters selectively facilitate the uptake of specific hydrophilic low-molecular-weight substrates via the alternating-access model, though their substrate selectivity is not always strict [15]. In contrast, nanoparticles are too large to traverse transporter pores and typically enter cells through endocytosis [16]. At the chemical level, nucleophiles attack electron-deficient electrophilic centers—a fundamental mechanism underlying enzyme and ribozyme catalysis—commonly represented using curved-arrow notation. According to frontier molecular orbital (FMO) theory [17] and the Woodward–Hoffmann rules [18], chemical bond formation involves interactions between the highest occupied molecular orbital (HOMO) and lowest unoccupied molecular orbital (LUMO). Electronic effects influencing reactivity are described by the Hammett equation [19]. According to the hard and soft acids and bases (HSAB) theory [20], hard acids preferentially interact with hard bases, whereas soft acids preferentially interact with soft bases. Thus, the behavior of compounds—characterized by molecular size, functional groups, and hydrophobic/hydrophilic balance—is governed by intrinsic physicochemical principles operating within biological systems. These principles regulate carrier-mediated transport, receptor-mediated endocytosis, and the central dogma processes of transcription and translation. This framework, reflecting structuralism proposed by Claude Lévi-Strauss [21,22], emphasizes that biological phenomena are structured and rule-based. Accordingly, rational modification of compounds by incorporating SLC transporter recognition units could intentionally confer membrane permeability. Highly rational, structure-based design approaches—akin to a “structuralism-conscious” Quality by Design (QbD) concept—may therefore achieve the desired pharmacokinetic and pharmacodynamic outcomes. Biological responses are fundamentally governed by chemical reactions, and it is considered that a molecular-level, theory-driven understanding is indispensable for addressing current challenges in pharmaceutical development. In the present situation, where the successful market introduction of new drug products has become increasingly difficult, it is believed that perspectives grounded in fundamental chemical principles—rather than conventional formulation-centered approaches alone—are critically needed.

**Figure 3:**
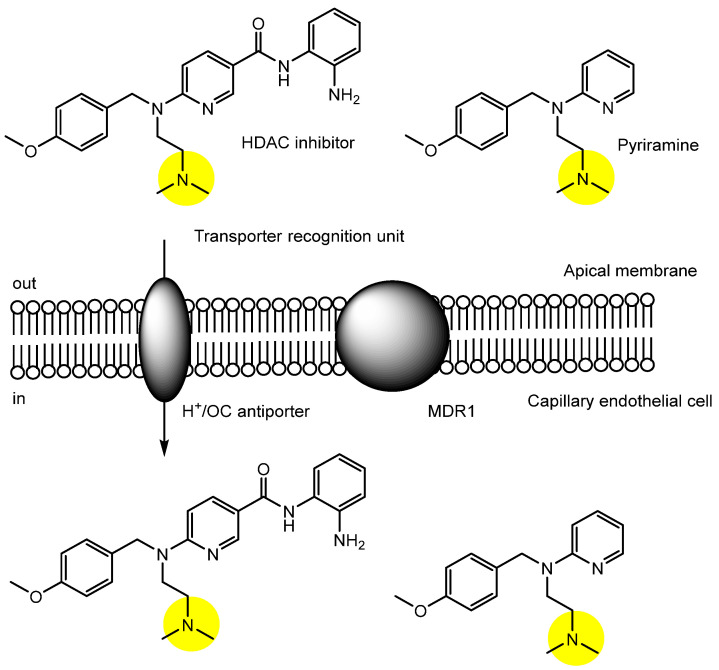
Mechanism of transport into capillary endothelial cells mediated by the proton-coupled organic cation (H^+^/OC) antiporter for a pyrilamine derivative containing an *N*-containing group and acting as a histone deacetylase (HDAC) inhibitor. Yellow circles indicate the transporter recognition units.

(i) It is well known that antihistamine drugs such as pyrilamine (Figure 3), most of which contain nitrogen-bearing functional groups, can cross the BBB and elicit off-target effects such as drowsiness [23]. In general, amines are protonated and carry a positive charge at physiological pH, as exemplified by memantine, which also possesses an *N*-containing group [24]. Consequently, positively charged amines under physiological conditions cannot readily cross the plasma membrane via passive diffusion. However, amine transporters—such as the proton-coupled organic cation (H^+^/OC) antiporter—facilitate the transmembrane transport of compounds containing *N*-functional groups [25,26]. A pyrilamine derivative bearing a benzamide zinc-binding moiety, designed as a HDAC inhibitor, was shown to be taken up into the brain microvascular endothelial cell line hCMEC/D3 via H^+^/OC antiporter-mediated transport in an in vitro assay. Furthermore, this compound successfully crossed the BBB in an in situ brain perfusion assay in rats (Figure 3) and demonstrated HDAC1 inhibitory activity [27]. Since SLC transporters are expressed in a tissue-specific manner, conjugates containing suitable transporter recognition units may be transported across the membranes of target cells—such as capillary endothelial cells at the BBB—via the H^+^/OC antiporter.

In summary, the H^+^/OC antiporter facilitates the transport of *N*-containing compounds across the BBB. A pyrilamine derivative, designed as an HDAC inhibitor, successfully crossed the BBB in rats using this mechanism.

Designs of conjugates incorporating transporter recognition units can generally be classified into two categories: (a) those in which the transporter recognition unit is integrated within the pharmacophore, and (b) those in which the transporter recognition unit is spatially separated from the pharmacophore [28]. The former case is exemplified by the pyrilamine derivative containing a benzamide zinc-binding group described above. The latter case involves conjugates in which transporter recognition units are introduced into the molecule as prodrugs via cleavable covalent linkages (e.g., ester bonds) or cleavable non-covalent static interactions, as well as through non-cleavable covalent bonds such as C–C linkages. Representative transporter recognition units include glucose moieties, which are recognized by glucose transporters such as glucose transporter 1 (GLUT1) [29], and peptide moieties, which are recognized by peptide transporter 1 (PEPT1) [30].

### 2.2. Conjugates with Ligands

**Figure 4:**
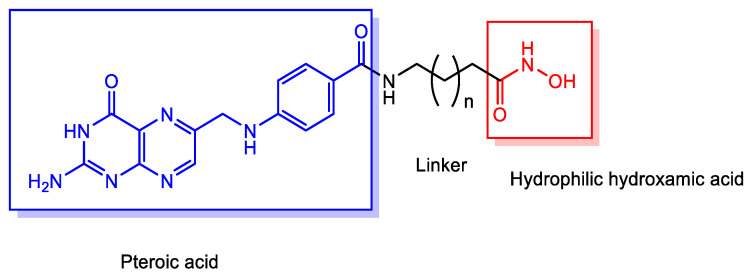
Chemical structures of conjugates between pteroic acid and hydroxamic acid linked via spacers of varying lengths (*n* = 1–7), designed as histone deacetylase (HDAC) inhibitors.

**Figure 5:**
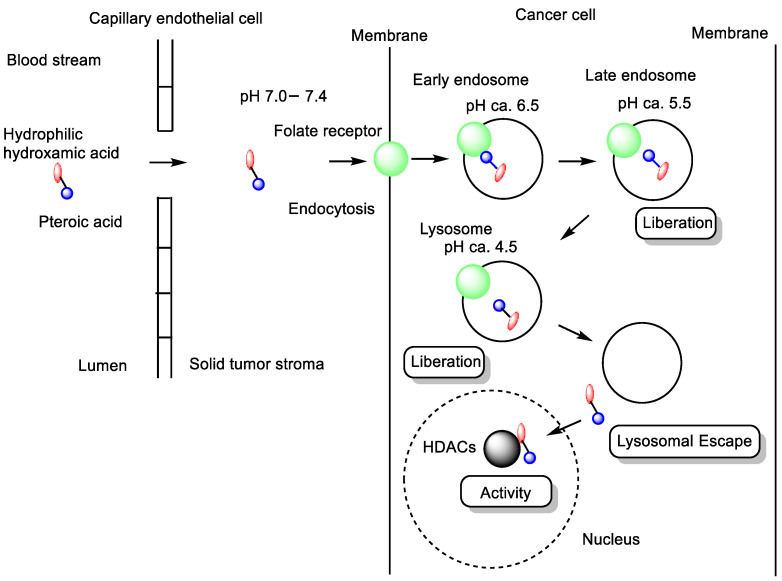
Schematic representation of the pathway of conjugates between pteroic acid (shown as blue circles) and hydroxamic acid (shown as red ellipses) linked via spacers. Conjugates internalized into cancer cells via receptor-mediated endocytosis using folate receptors (shown as green circles) and interacted with histone deacetylases (HDACs) (shown as black circles) after ensosomal or lysosomal escape.

**Figure 6:**
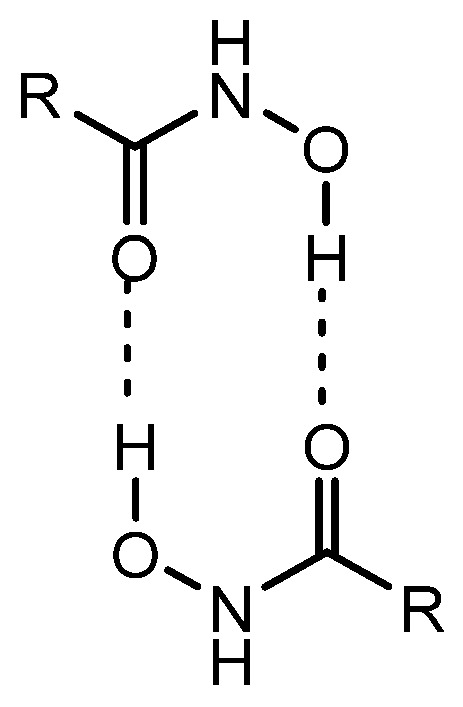
Chemical structures of hydroxamic acid dimers. R denotes variable side chains.

Drug delivery can also be achieved by using ligand-conjugated compounds as vectors. Endocytosis is a cellular uptake process in which portions of the plasma membrane, together with its constituent proteins such as receptors and lipids, are internalized to form endosomes through membrane invagination. This process allows the cell to engulf extracellular materials, including ligands and bystander solutes. Mechanistically, endocytosis based on the mode of plasma membrane invagination is categorized into (a) clathrin-dependent endocytosis (endosomal diameter: 85–150 nm); (b) caveolae-dependent endocytosis (50–100 nm); (c) clathrin- and caveolae-independent endocytosis (approximately 90 nm); (d) macropinocytosis (0.2–5 μm); and (e) other less well-understood or mechanically complex forms of endocytosis [14]. Several endocytic mechanisms may occur simultaneously within the same cell, although clathrin-mediated endocytosis is generally the predominant pathway. In addition, endocytosis can be classified according to its triggering mechanism as: (a) receptor-mediated endocytosis, (b) non-receptor-mediated endocytosis, and (c) caveolae/lipid raft-mediated endocytosis, which is often considered a subtype of receptor-mediated endocytosis because it involves cholesterol-rich microdomains that concentrate specific receptors [14]. The term “adsorptive endocytosis” is sometimes used to describe receptor-mediated endocytosis involving ubiquitous negatively charged heparan sulfate proteoglycans (HSPGs), which can nonspecifically attract and internalize cationic molecules through electrostatic interactions.

(ii) Folic acid, consisting of pteroic acid linked to a glutamate residue, is a member of the vitamin B complex that plays essential roles in cell growth, reproduction, and DNA synthesis [31]. Folate receptors (FRs) are frequently overexpressed on the surface of various cancer cells and mediate the endocytic uptake of folate-conjugated compounds. In contrast, reduced folate carriers (RFCs), which belong to the SLC transporter family, facilitate the transport of native folates—but not folate conjugates—into normal cells [32,33]. Therefore, HDAC inhibitors conjugated with pteroic acid (e.g., *n* = 5 spacer units) may be selectively delivered into cancer cells via FR-mediated endocytosis. Indeed, a folate-based hydroxamate HDAC inhibitor (Figure 4) exhibited potent anticancer activity against FR-positive KB (oral carcinoma) and HeLa (cervical carcinoma) cells, both of which are FR-overexpressing cell lines, and also demonstrated low-nanomolar HDAC1 inhibitory potency. In contrast, the conjugate showed no significant activity against the FR-negative A549 lung carcinoma cell line [34]. Pharmacokinetic analyses indicated that its cellular uptake proceeded via receptor-mediated endocytosis through FRs rather than via carrier-mediated transport by RFCs. The subsequent endosomal escape was proposed to occur through mechanisms such as membrane disruption or surfactant-like effects, rather than by passive diffusion, since the compound displayed poor plasma membrane permeability in FR(−) lung cancer A549 cells. It is well known that ligand clustering on the cell surface can induce endocytosis [35,36,37]. Accordingly, hydroxamate dimers with long linkers (Figure 5) may enhance FR clustering—a multiligand strategy that promotes endocytic uptake. Conjugates with short carbon linkers (*n* = 1–2) failed to show cytotoxic or HDAC-inhibitory activity in KB and HeLa cells, whereas conjugates with longer linkers (*n* = 3–7) retained both activities [34]. These findings suggest that cluster formation may play a key role in the endocytosis of HDAC inhibitors conjugated with pteroic acid moieties. Moreover, the higher activity of conjugates with longer linkers (*n* = 3–7) might also be attributed to improved pharmacodynamic complementarity between the conjugate and the HDAC active site cavity, consistent with both the key–lock and induced-fit theories of molecular recognition. Additionally, their increased hydrophobicity could potentially facilitate endosomal escape via passive diffusion or local membrane disruption (Figure 6). However, this hypothesis is contradicted by the observation that the conjugate with *n* = 5 exhibited poor plasma membrane permeability in FR(−) A549 cells, indicating that FR-mediated endocytosis, rather than hydrophobic diffusion, is the dominant uptake pathway.

Consequently, folate-conjugated HDAC inhibitors can selectively target cancer cells overexpressing FRs through receptor-mediated endocytosis, with longer multiligand linkers potentially enhancing uptake and activity by promoting FR clustering and improving drug-target fit. Despite increased hydrophobicity, FR-mediated endocytosis, not passive diffusion, is the primary uptake mechanism for these compounds.

### 2.3. Conjugates with CPPs

**Figure 7:**
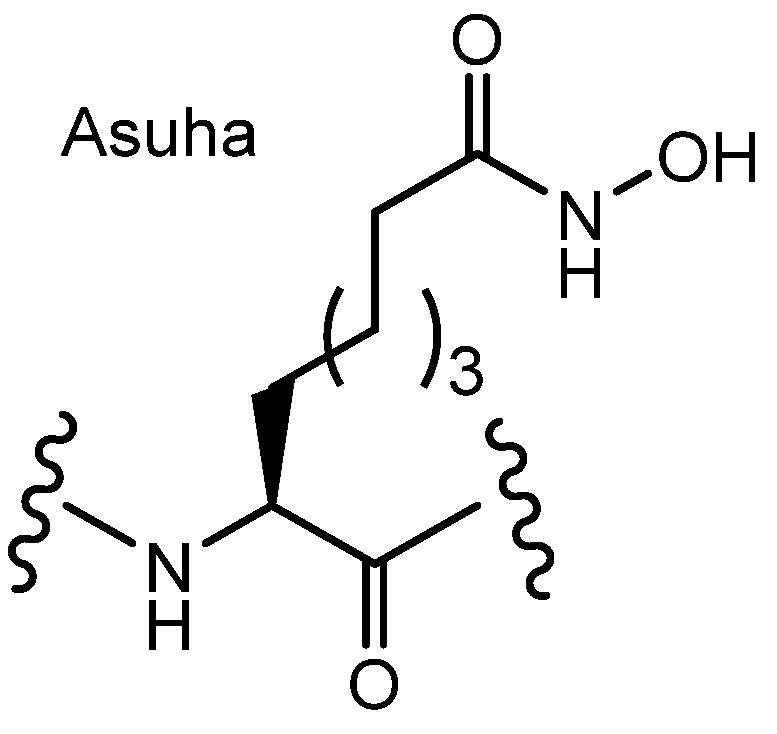
Structures of hydroxamic acid-containing aminosuberic acid (Asuha) analogs.

Drug delivery can also be accomplished through conjugation with CPPs as vectors. CPPs are short cationic peptides, typically consisting of 5–30 amino acids, that can penetrate cellular membranes via endocytosis or direct translocation. Representative CPPs include TAT (YGRKKRRQRRR), penetratin (RQIKIWFQNRRMKWKK), and octaarginine (R8). Many CPPs interact electrostatically with negatively charged HSPGs, thereby triggering receptor-mediated endocytosis [38]. In contrast, certain CPPs are thought to promote lipid raft-mediated endocytosis, which can also be considered a form of receptor-mediated endocytosi due to its dependence on temperature and peptide concentration [39]. Consequently, CPPs have been widely utilized as vectors for drug delivery [40]. However, only a few conjugates combining CPPs with epigenetic drugs have been reported, possibly due to the unfavorable size disparity between the small-molecule drug and the relatively large peptide vector.

(iii) HDAC isozymes are categorized into two major groups: zinc-dependent metallohydrolases, which include class I (HDACs 1–3 and 8), class IIa (HDACs 4, 5, 7, and 9), class IIb (HDACs 6 and 10), and class IV (HDAC11); and NAD^+^-dependent protein deacetylases, comprising class III (sirtuins 1–7) [41]. These enzymes are expressed in a tissue- and cell type-specific manner. Hydroxamic acid-containing aminosuberic acid (Asuha) peptide analogs (Figure 7) have been shown to inhibit class I HDAC isozymes in microarray assays. Treatment with hydroxamic acid-modified peptides such as H4(5–19)K12Asuha (Ac-KGGKGLGXGGAKRHR-W-NH_2_, X = Asuha, 10 µM) and H3(7–22)K18Asuha (Ac-ARKSTGGKAPRXQLAT-W-NH_2_, X = Asuha, 10 µM) induced acetylation of α-tubulin and histone residues (H3K27, H3K36) in HEK293T cells after 5 h of incubation, as demonstrated by Western blot analysis, compared with the DMSO-treated control [42]. These results indicate that the peptides were capable of entering cells in a manner similar to CPPs. Because peptides generally cannot cross the plasma membrane by passive diffusion due to their hydrophilicity and size, it is likely that Asuha-containing peptide inhibitors are internalized via receptor-mediated endocytosis. The H4(5–19)K12Asuha peptide carries a slight positive charge owing to its three lysine and two arginine residues, and the H3(7–22)K18Asuha peptide likewise exhibits a mild positive charge from its two lysine and two arginine residues. Therefore, these peptides may enter cells through receptor-mediated endocytosis involving negatively charged HSPGs—a mechanism that could be competitively inhibited by heparin if added to the system.

Thus, hydroxamic acid-containing Asuha peptides act as class I HDAC inhibitors and demonstrate effective intracellular delivery, leading to increased acetylation of histones and α-tubulin in cells. Their mild positive charge likely facilitates cellular uptake via HSPG-mediated receptor-dependent endocytosis, highlighting their potential as peptide-based drug delivery agents for intracellular epigenetic targets.

### 2.4. Conjugates with Homing Peptides

**Figure 8:**
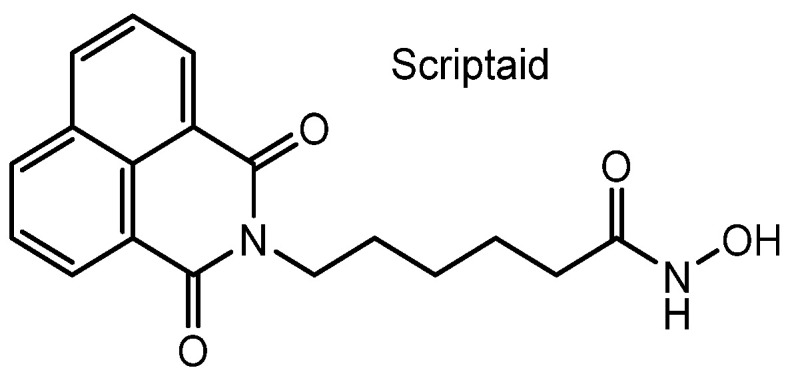
Chemical structure of scriptaid, a histone deacetylase (HDAC) inhibitor.

The use of homing peptide-conjugated systems also enables targeted drug delivery to cancer cells. The Arg–Gly–Asp (RGD) sequence, a well-known THP, binds to αVβ3 and αVβ5 integrins and subsequently induces biological events such as endocytosis, cell migration, growth, differentiation, and apoptosis [43]. The Asn–Gly–Arg (NGR) sequence, another representative THP, binds to the receptor aminopeptidase N (CD13) [43]. Therefore, conjugates incorporating THPs may be internalized into cancer cells via receptor-mediated endocytosis.

(iv) Delta24-RGD, a genetically engineered tumor-selective adenovirus, targets αVβ3 and αVβ5 integrins through an inserted RGD motif and consequently enters cancer cells, particularly glioblastomas. When combined with HDAC inhibitors such as panobinostat (LBH589) (Figure 1) or scriptaid (Figure 8), Delta24-RGD exhibited enhanced antitumor activity in in vitro assays using patient-derived glioblastoma stem-like cells, compared with either monotherapy alone [44]. It is likely that panobinostat and scriptaid were co-internalized as bystander molecules through Delta24-RGD-induced endocytosis. However, in in vivo conditions, HDAC inhibitors and Delta24-RGD may become separated during systemic distribution, thereby diminishing HDAC inhibitory effects if the drugs fail to enter cancer cells as bystanders of Delta24-RGD-mediated uptake.

In short, the tumor-targeting adenovirus Delta24-RGD enhances intracellular delivery of HDAC inhibitors by inducing integrin-mediated endocytosis, leading to synergistic antitumor effects in glioblastoma cells in vitro. However, in vivo efficacy may be limited because HDAC inhibitors can dissociate from the virus during systemic circulation, reducing their bystander uptake into cancer cells.

### 2.5. Conjugates with Monoclonal Antibodies

Monoclonal antibodies exhibit remarkably high selectivity toward their target antigens compared with CPPs and THPs. Drug delivery employing monoclonal antibodies as molecularly targeted agents is expected to minimize off-target side effects through active targeting. Furthermore, monoclonal antibodies, such as the IgG molecule (approximately 14.2 nm in diameter [45]), can also accumulate in tumor tissues via passive targeting mediated by the enhanced permeability and retention (EPR) effect [46]. This effect, primarily associated with nanoparticles ranging from 10 to 100 nm in diameter, arises from the leaky vasculature and impaired lymphatic drainage characteristic of tumors. Therefore, the antibody–drug conjugate (ADC) strategy represents a highly promising approach for selective drug delivery.

**Figure 9:**
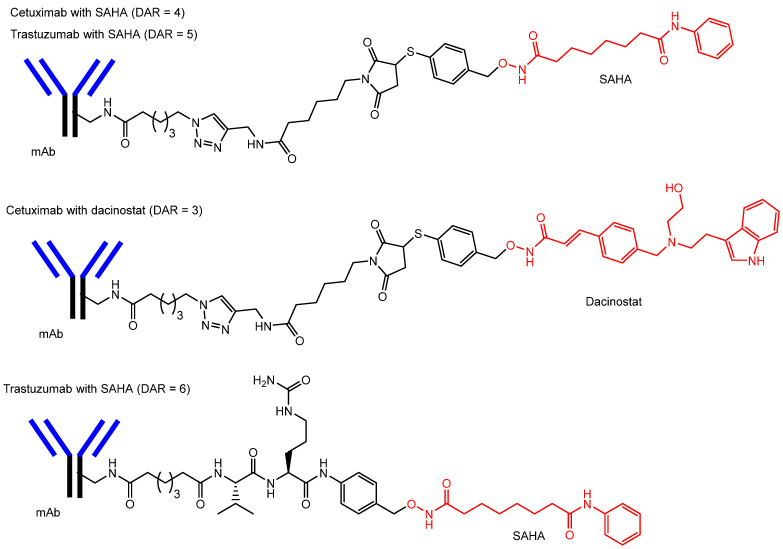
Chemical structures of antibody–drug conjugates, including cetuximab–SAHA (DAR = 4), trastuzumab–SAHA (DAR = 5), cetuximab–dacinostat (DAR = 3), and trastuzumab–SAHA (DAR = 6), linked through arbitrary linkers. The drug-to-antibody ratio (DAR) was determined by MALDI mass spectrometric analysis. Antibodies are indicated in blue and black, whereas histone deacetylase (HDAC) inhibitors are indicated in red.

**Figure 10:**
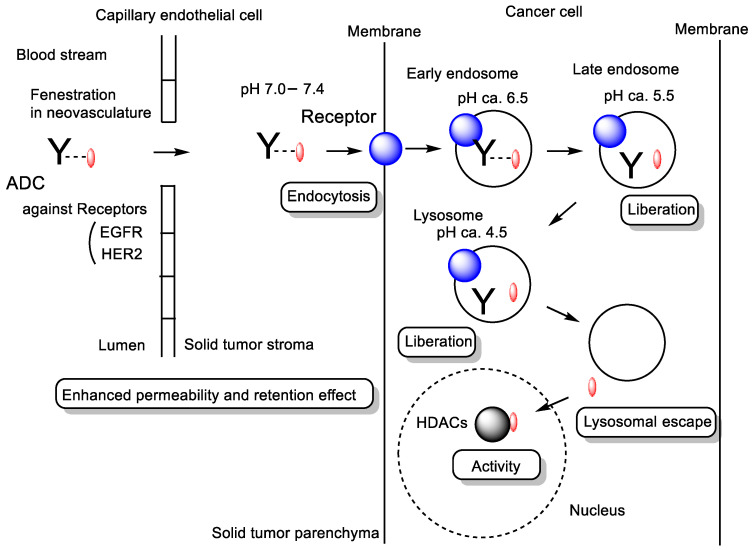
Proposed pathway of antibody–drug conjugates (ADCs) penetrating the solid tumor stroma via the enhanced permeability and retention (EPR) effect, which arises from the leaky nature of tumor vasculature. ADCs are subsequently internalized into cancer cells through receptor-mediated endocytosis involving receptors (shown as blue circles) such as the epidermal growth factor receptor (EGFR) or human epidermal growth factor receptor 2 (HER2). The conjugated drugs, such as SAHA or dacinostat (shown as red ellipses), are released from endosomes or lysosomes and ultimately inhibit histone deacetylases (HDACs) (shown as black circles). Y denotes a monoclonal antibody.

(v) Indeed, SAHA and dacinostat conjugated to cetuximab and trastuzumab through a non-cleavable linker (Figure 9) inhibited HDAC activity in several tumor cell lines, respectively [47]. Monoclonal antibodies, including ADCs, tend to accumulate spontaneously in tumor tissues via the EPR effect. Cetuximab is a monoclonal antibody targeting the epidermal growth factor receptor (EGFR) [48], whereas trastuzumab targets the human epidermal growth factor receptor 2 (HER2) [49]. These antibodies bind to their respective cancer-associated antigens, EGFR or HER2, on the surface of tumor cells and subsequently trigger receptor-mediated endocytosis. Within endosomes or lysosomes, hydroxamate groups are liberated during endosomal maturation, and the released drug molecules can then diffuse into the cytosol, most likely via passive diffusion (Figure 10). Lysosomes contain a variety of degradative enzymes capable of cleaving ADC linkers, while some linkers may also undergo hydrolysis under the acidic lysosomal environment.

**Figure 11:**
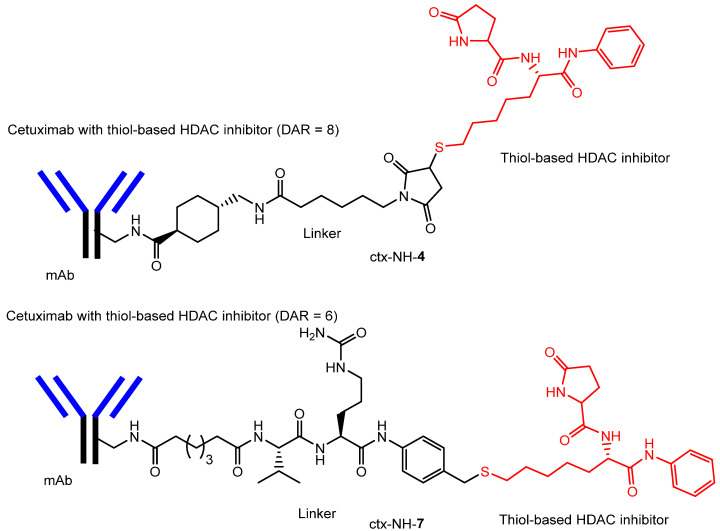
The structures of antibody–drug conjugates (ADCs), including cetuximab conjugated with a thiol-based histone deacetylase (HDAC) inhibitor (DAR = 8; ctx-NH-**4**) and cetuximab conjugated with a thiol-based HDAC inhibitor (DAR = 6; ctx-NH-**7**), linked through arbitrary spacers. The drug-to-antibody ratio (DAR) was determined by MALDI mass spectrometric analysis. Antibodies are indicated in blue and black, whereas histone deacetylase (HDAC) inhibitors are indicated in red.

As a result, conjugation of HDAC inhibitors such as SAHA and dacinostat to tumor-targeting antibodies (cetuximab or trastuzumab) enables selective accumulation in tumors and receptor-mediated endocytosis via EGFR or HER2. Following lysosomal processing, the HDAC inhibitors are released intracellularly and diffuse into the cytosol, allowing effective HDAC inhibition with enhanced tumor specificity.

(vi) Moreover, the thiol-based HDAC inhibitor exhibited half-maximal inhibitory concentration (IC_50_) values of 13, 5, 3, and 11 nM against HDAC1, HDAC3, HDAC6, and HDAC10, respectively, and an IC_50_ value of 0.07 μM against the lung cancer cell line NCI-H460. The conjugate of this thiol-based HDAC inhibitor with cetuximab (ctx-NH-**4**) (Figure 11) inhibited the proliferation of two lung adenocarcinoma cell lines, NCI-H1975 and Calu-3, showing an IC_50_ of 250 nM in NCI-H1975 cells. Another conjugate, cetuximab–thiol-based HDAC inhibitor (ctx-NH-**7**) (Figure 11), demonstrated significantly greater antitumor activity against NCI-H1975 cells compared with cetuximab alone targeting EGFR [50]. In the short term, intracellular HDAC inhibition produced more potent anticancer effects than extracellular EGFR inhibition. The pharmacokinetic profile and overall activity were improved by conversion to an ADC format [50].

Therefore, conjugation of a highly potent thiol-based HDAC inhibitor to cetuximab enables EGFR-targeted delivery and efficient intracellular HDAC inhibition, resulting in stronger anticancer activity than EGFR blockade alone. Conversion of the inhibitor into an ADC format improves pharmacokinetics and tumor cell uptake, thereby enhancing overall therapeutic efficacy.

### 2.6. Conjugates with Nanoparticles

Despite frequent concerns regarding the limited clinical translation of nanomedicine, it should be emphasized that multiple nanoparticle-based formulations have already received regulatory approval and are routinely used in oncology. Representative examples include pegylated liposomal doxorubicin (Doxil^®^) for ovarian cancer and AIDS-related Kaposi’s sarcoma, albumin-bound paclitaxel nanoparticles (Abraxane^®^) for breast, lung, and pancreatic cancers, liposomal irinotecan (Onivyde^®^) for pancreatic adenocarcinoma, and the fixed-ratio liposomal combination of daunorubicin and cytarabine (Vyxeos^®^) for high-risk acute myeloid leukemia [51]. Notably, several of these products have demonstrated clear clinical benefits, including improved pharmacokinetics, reduced systemic toxicity, and, in some cases, survival advantages, with ongoing clinical studies further expanding their indications. These precedents clearly indicate that nanoparticle-based drug delivery systems are not limited to preclinical proof-of-concept but have achieved meaningful clinical and translational success. Accordingly, it is reasonable to anticipate that epigenetic drugs—many of which currently face challenges related to bioavailability, off-target toxicity, and pharmacokinetics—may similarly benefit from rational nanoparticle-enabled delivery strategies, facilitating their advancement toward clinical application.

**Figure 12:**
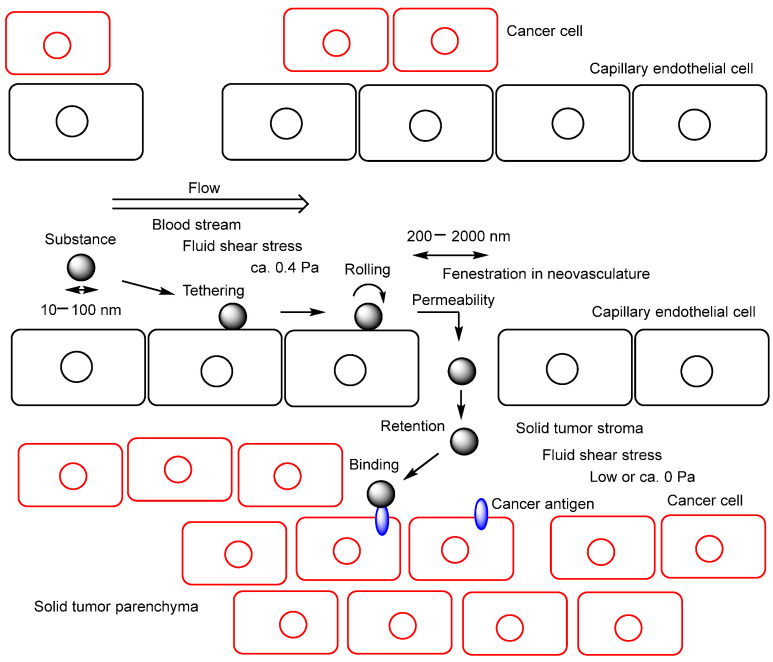
Mechanism of the enhanced permeability and retention (EPR) effect. Substances ranging from 10 to 100 nm in diameter—such as monoclonal antibodies and nanoparticles—can spontaneously accumulate within the stroma or parenchyma of solid tumors through fenestrations (200–2000 nm wide) in the capillary endothelium of tumor neovasculature. These fenestrations arise from vascular endothelial growth factor (VEGF)-induced angiogenesis and are associated with reduced vascular integrity and wall fluid shear stress (FSS) of approximately 0.4 Pa. Once extravasated, these substances remain within the tumor microenvironment due to the underdeveloped lymphatic drainage system and the elevated interstitial fluid pressure characteristic of solid tumor tissue.

Nanomaterial-based drug delivery systems have significantly advanced the technological development of safer and more therapeutically effective treatments. Nanoparticles can be utilized not only to protect siRNAs and antisense oligonucleotides from enzymatic degradation but also to deliver epigenetic anticancer agents to tumor cells through passive targeting of cancer tissues via the EPR effect [46]. The EPR effect is a pathophysiological phenomenon that enables nanoparticles (typically 10–100 nm in diameter) to accumulate within the stroma and parenchyma of solid tumors due to the leaky nature of tumor vasculature, the underdeveloped lymphatic drainage system, and the elevated interstitial fluid pressure in the tumor microenvironment [52,53] (Figure 12). Furthermore, nanoparticles functionalized with CPPs, THPs, or monoclonal antibodies can achieve active targeting in addition to passive targeting based on the EPR effect.

(vii) Tannic acid (TA), a polyphenolic compound (MW = 1701.20), exhibits anticancer properties but suffers from low bioavailability [54]. A nanodelivery system has been explored as a potential solution. TA-loaded chitosan-based nanoparticles (Chi-TA-NPs), with an average diameter of 567.0 ± 25.84 nm, released approximately 44% of their TA content after 300 min [54]. In vitro cytotoxicity assays using human HepG2 hepatocellular carcinoma (HCC) cells demonstrated that Chi-TA-NPs exerted greater cytotoxic effects than free TA, associated with altered global DNA methylation and differential expression of DNMTs. The expression levels of DNMT1, DNMT3A, and DNMT3B were quantified by RT-qPCR, revealing that Chi-TA-NPs reduced the expression of DNMT1 (2.52-fold), DNMT3A (2.96-fold), and DNMT3B (2.94-fold) compared with TA alone [54]. The precise mechanisms of Chi-TA-NP cellular internalization remain unclear, although endocytosis is likely to be involved. Moreover, the molecular mechanisms underlying TA’s anticancer activity are not yet fully understood. Given their relatively large particle size (>500 nm), Chi-TA-NPs are likely too large for receptor-mediated endocytosis and may instead enter cells primarily through macropinocytosis.

**Figure 13:**
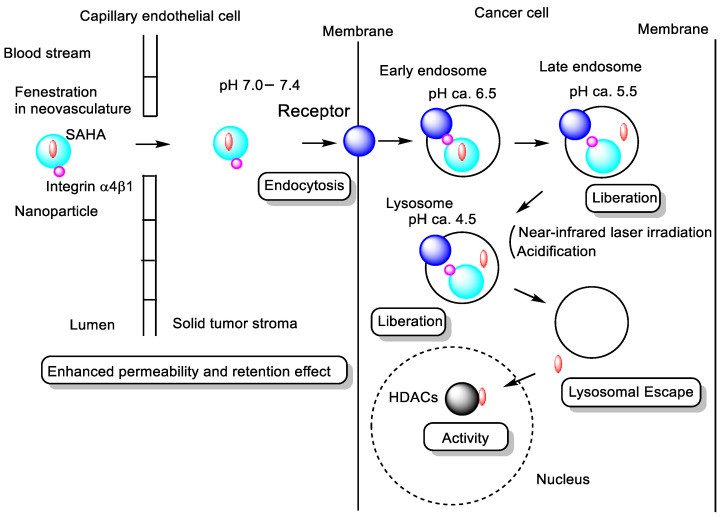
Schematic representation of the pathway of suberoylanilide hydroxamic acid (SAHA)-encapsulated black phosphorus nanoparticles (depicted as light blue circles) camouflaged with M1 macrophage membranes expressing integrin α4β1 (depicted as magenta circles) (MBS). The MBS accumulate preferentially in solid tumor tissues via the enhanced permeability and retention (EPR) effect and are subsequently internalized into cancer cells through receptor-mediated endocytosis involving tumor-associated receptors (depicted as blue circles). Upon near-infrared (NIR) laser irradiation, SAHA molecules (depicted as red ellipses) are released from the black phosphorus nanoparticles and diffuse from endosomes into the cytosol and nucleus. There, SAHA inhibits histone deacetylases (HDACs) (depicted as black circles), resulting in anti-cancer effects.

Thus, encapsulation of tannic acid into chitosan-based nanoparticles improves its bioavailability and intracellular delivery, resulting in enhanced cytotoxicity and epigenetic modulation in hepatocellular carcinoma cells compared with free tannic acid. Due to their large particle size, these nanoparticles are likely internalized mainly through macropinocytosis, highlighting nanoparticle-mediated uptake as a viable delivery strategy for poorly bioavailable epigenetic drugs.

(viii) SAHA-loaded black phosphorus nanoparticles camouflaged with M1 macrophage membranes (MBS) (ca. 100 nm in diameter) exhibited potent anti-tumor efficacy in an in vivo lung cancer xenograft mouse model under near-infrared (NIR) laser irradiation [55]. The M1 macrophage membrane coating enhanced tumor-targeting specificity, attributed to the expression of integrin α4β1 on the macrophage surface [54]. The internalization of MBS by tumor cells is presumed to occur via receptor-mediated endocytosis involving receptors that recognize integrin α4β1. Black phosphorus nanoparticles are photoresponsive to NIR laser exposure, enabling controlled drug release within endosomes. Subsequently, SAHA diffuses passively from endosomes into the cytosol and nucleus, where it exerts HDAC inhibitory activity in lung cancer cells (Figure 13). Moreover, the macrophage membrane coating confers a stealth effect, extending the nanoparticles’ circulation time by reducing opsonization and phagocytic clearance by the reticuloendothelial system [55].

**Figure 14:**
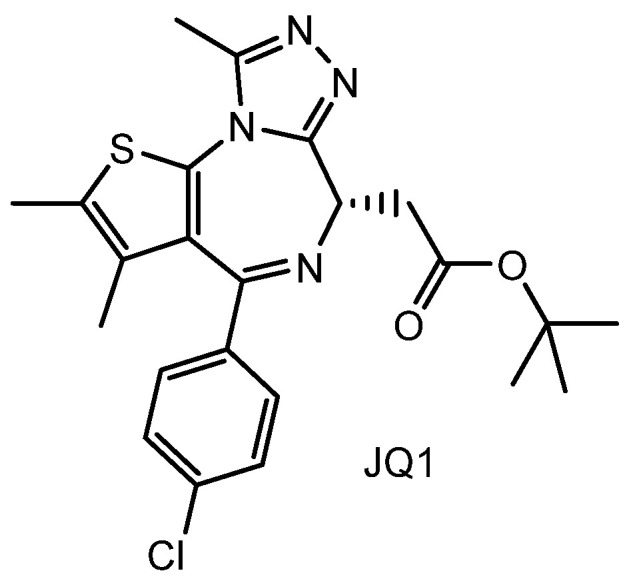
Chemical structure of the BET bromodomain inhibitor JQ1.

Therefore, camouflaging SAHA-loaded black phosphorus nanoparticles with M1 macrophage membranes enhances tumor targeting and circulation time, enabling integrin α4β1-mediated endocytosis into lung cancer cells. Upon NIR irradiation, the photoresponsive nanoparticles release SAHA intracellularly, allowing effective HDAC inhibition through controlled endosomal-to-cytosolic drug delivery.

(ix) JQ1 is a bromodomain and extraterminal domain (BET) inhibitor (Figure 14). Hypoxia-cleavable, RGD peptide-modified poly(D,L-lactide-co-glycolide) (PLGA) nanoparticles (ARNPs) loaded with JQ1 (108.39 ± 5.82 nm in diameter) effectively suppressed tumor growth by 65.8% compared with the 5% glucose control group in 4T1 cell-xenografted mice. Moreover, JQ1-loaded ARNPs inhibited pulmonary metastasis secondary to bone metastasis in the same mouse model [56]. Although the RGD peptides were initially shielded by PEG chains, the hypoxic tumor microenvironment triggered azobenzene cleavage of the PEG layer, thereby exposing the RGD motifs to interact with αvβ3 integrins and initiate receptor-mediated endocytosis [56]. The JQ1-loaded ARNPs likely accumulated in hypoxic tumor tissues through the EPR effect, followed by active targeting via the RGD–αvβ3 integrin interaction. Consequently, this dual-targeting mechanism minimizes off-target side effects by combining both passive and active targeting strategies [56]. The ARNP-based drug delivery platform may also be applicable to other anti-cancer agents such as paclitaxel, which targets tubulin, and may serve as an effective system for combination therapies involving multiple anticancer agents.

Consequently, hypoxia-responsive, RGD-modified PLGA nanoparticles enable efficient tumor-targeted delivery of the BET inhibitor JQ1 by combining EPR-mediated passive accumulation with hypoxia-triggered exposure of RGD ligands for αvβ3 integrin-mediated endocytosis. This dual-targeting strategy enhances antitumor and antimetastatic efficacy while reducing off-target effects, highlighting the versatility of ARNPs as a controlled nanodelivery platform for epigenetic therapeutics.

**Figure 15:**
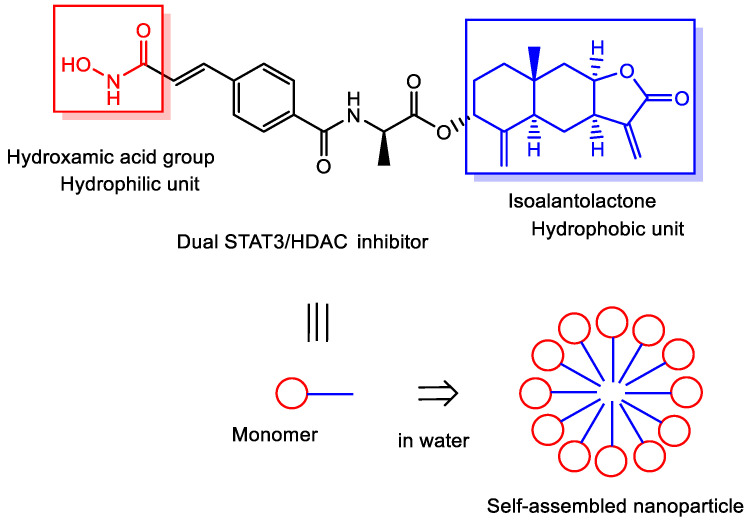
Self-assembled nanoparticles composed of a dual inhibitor targeting signal transducer and activator of transcription 3 (STAT3) and histone deacetylases (HDACs). Isoalantolactone, a hydrophobic sesquiterpene lactone, exerts its anti-cancer effects primarily through inhibition of STAT3 signaling.

(x) A highly potent dual inhibitor targeting both signal transducer and activator of transcription 3 (STAT3) and HDACs, structurally derived from isoalantolactone conjugated with a hydroxamic acid moiety, was shown to self-assemble in aqueous solution to form nanoparticles (Figure 15). Interestingly, these self-assembled nanoparticles accumulated preferentially in tumor tissues via the EPR effect, promoted cellular uptake, and consequently exhibited a more potent anti-cancer effect as dual inhibitors compared with their non-self-assembled counterparts. Moreover, this dual inhibitor demonstrated greater anti-cancer efficacy than either SAHA or isoalantolactone alone in both in vitro and in vivo assays [57]. The self-assembled nanoparticles were likely to disaggregate into monomeric units within the cytosol following transmembrane transport, although the detailed mechanism remains unclear. Structurally, the dual inhibitor consists of a hydrophobic isoalantolactone unit and a hydrophilic hydroxamic acid group, resembling the amphiphilic nature of surfactants or lipids. Therefore, some dissociated monomers at the plasma membrane may insert into the lipid bilayer, undergo translocation (“flip-flop”), and subsequently migrate into the cytosol. Alternatively, the nanoparticles may fuse with the plasma membrane or enter cells via lipid raft-mediated or other forms of endocytosis. Furthermore, the self-assembled nanoparticles are considered to exhibit minimal cytotoxicity due to steric hindrance, despite the hydroxamic acid group being partially exposed on the nanoparticle surface. In contrast, the non-self-assembled equivalents would likely show increased nonspecific toxicity and reduced anti-cancer efficacy, as they cannot benefit from EPR-based tumor accumulation.

In short, an amphiphilic dual STAT3/HDAC inhibitor self-assembles into nanoparticles that preferentially accumulate in tumors via the EPR effect, enhancing cellular uptake and antitumor efficacy compared with non-assembled molecules. This self-nanodelivery system likely enables intracellular drug release through membrane interaction or endocytic pathways while reducing nonspecific toxicity and improving therapeutic selectivity.

(xi) Decitabine (Figure 2), a DNMT inhibitor, has demonstrated significant efficacy in the treatment of leukemia [58]. However, its clinical application to solid tumors such as HCC is limited due to its short plasma half-life. Nanoparticle-based delivery systems can potentially extend the half-life of decitabine by protecting it from enzymatic degradation. Programmed death-ligand 1 (PD-L1) is expressed on the surface of many cancer cells, including liver cancer cells, whereas programmed cell death protein 1 (PD-1) is expressed on T cells [59]. The PD-1/PD-L1 signaling axis functions as an immune checkpoint that maintains self-tolerance but also facilitates tumor immune evasion [60]. To overcome these limitations, PLGA-based spherical nanoparticles encapsulating decitabine (Dec@PLGA) were coated with both an anti-PD-L1 antibody and a macrophage membrane (aMM) to form Dec@PLGA@aMM. These hybrid nanoparticles (95.7 ± 9.1 nm in diameter) significantly enhanced tumor suppression by (a) reactivating tumor suppressor genes epigenetically silenced by DNA hypermethylation and (b) blocking PD-L1 through antibody-mediated immune checkpoint inhibition [61]. Dec@PLGA@aMM is believed to enter cancer cells via receptor-mediated endocytosis, potentially through interactions between integrins on macrophage membranes and corresponding receptors on tumor cells, or between PD-L1 and the surface-bound anti-PD-L1 antibodies. Expression levels of the tumor suppressor proteins p14 and p16 were upregulated in a concentration-dependent manner following Dec@PLGA@aMM treatment. Notably, loss of p14 expression has been reported to reduce tumor immunogenicity in melanoma [62].

Accordingly, hybrid nanoparticles composed of decitabine-loaded PLGA coated with anti-PD-L1 antibodies and macrophage membranes enhance decitabine stability, tumor targeting, and cellular uptake via receptor-mediated endocytosis. This integrated delivery system combines epigenetic reactivation of tumor suppressor genes with immune checkpoint blockade, resulting in improved antitumor efficacy against hepatocellular carcinoma.

(xii) The ten–eleven translocation (TET) family of proteins catalyzes the oxidation of 5-methylcytosine in DNA, thereby initiating the active DNA demethylation process. Suppression of TET1 expression is a characteristic feature of colorectal cancer, and the promoter CpG island of the *TET1* gene is frequently hypermethylated in various malignancies [63]. PLGA core nanoparticles encapsulating decitabine and hybridized with TET1 gene-encoding plasmid DNA (pDNA) on the lipid shell surface (ca. 200 nm in diameter) effectively upregulated tumor suppressor gene expression and rapidly induced cell-cycle arrest in HCT116 human colon cancer cells [64]. The internalization mechanism of these PLGA core nanoparticles remains to be fully elucidated, although uptake is likely mediated by endocytosis or membrane fusion.

Thus, PLGA core nanoparticles co-delivering decitabine and TET1-encoding plasmid DNA enable combinatorial epigenetic therapy by restoring DNA demethylation and tumor suppressor gene expression in colorectal cancer cells. This nanocarrier system likely enters cells via endocytosis or membrane fusion, facilitating efficient intracellular delivery of both small-molecule and gene therapeutics.

**Figure 16:**
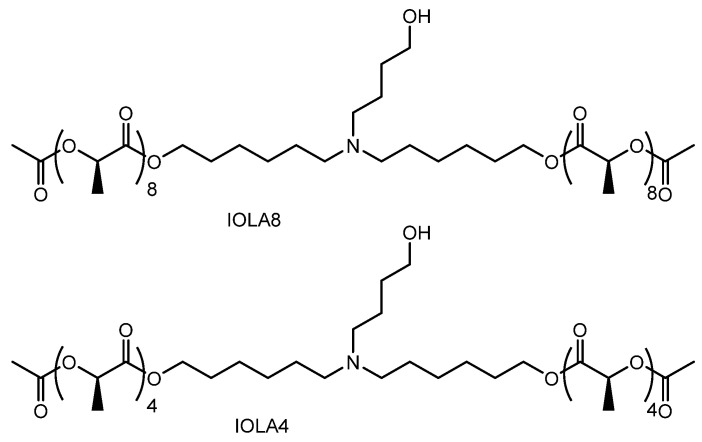
Chemical structures of ionizable oligolactic acid 4 (IOLA4) and ionizable oligolactic acid 8 (IOLA8), which constitute the ionizable polymeric micelles.

In addition to low-molecular-weight epigenetic modulators, miRNAs, siRNAs, and long non-coding RNAs (lncRNAs) also regulate epigenetic processes [65]. The integration of nanomedicine into cancer therapy enables the efficient delivery of these nucleic acid-based therapeutics, as encapsulation protects them from enzymatic degradation [66,67].

(xiii) Hepatic fibrosis is characterized by the excessive accumulation of extracellular matrix (ECM) proteins, such as collagen, primarily produced by activated hepatic stellate cells (HSCs) [68], and ultimately progresses to liver cirrhosis. Heat shock protein 47 (HSP47) is a collagen-specific molecular chaperone that promotes excessive collagen secretion [69]. High mobility group box 1 (HMGB1), secreted by various cells including damaged hepatocytes and macrophages, further activates Kupffer cells and induces the activation and proliferation of quiescent hepatic stellate cells [70]. Ionizable polymeric micelles (IPMs), composed of oligolactic acids with different tail lengths (*n* = 4 or 8), designated as IPM (IOLA4) (ca. 175 nm in diameter) and IPM (IOLA8) (ca. 140 nm in diameter) (Figure 16), were developed as delivery vehicles. Fibroblast activation protein inhibitor-modified ionizable polymeric micelles (FAPi-IPMs) encapsulating both siHSP47 and siHMGB1 reduced collagen secretion and hepatic inflammation in activated HSCs [71]. Fibroblast activation proteins (FAPs) are expressed on the surface of activated hepatic stellate cells, and thus FAPi-IPMs were internalized via receptor-mediated endocytosis through the FAPi–FAP interaction [71]. Following internalization, siHSP47 and siHMGB1 were released into the cytosol after endosomal or lysosomal escape. siHSP47 suppressed hepatic fibrosis by inhibiting collagen production, whereas siHMGB1 reduced hepatic inflammation and thereby attenuated fibrosis progression. The precise mechanisms underlying endosomal or lysosomal escape of siHSP47 and siHMGB1 remain unclear.

Hence, fibroblast activation protein inhibitor-modified ionizable polymeric micelles selectively deliver siHSP47 and siHMGB1 to activated hepatic stellate cells via FAP-mediated endocytosis. This targeted delivery enables cytosolic release of antifibrotic siRNAs, reducing collagen production and inflammation to effectively attenuate hepatic fibrosis progression.

(xiv) Myocardial infarction (MI), commonly known as a heart attack, is pathologically characterized by myocardial cell death caused by acute obstruction of a coronary artery. Abnormal DNA methylation patterns have been observed in myocardial infarction and other cardiovascular diseases, suggesting an association between DNA methylation and cardiovascular outcomes [72]. Azacitidine is a DNMT inhibitor (Figure 2). In human mesenchymal stem cells (hMSCs), miR-133a promotes cardiogenic differentiation by targeting epidermal growth factor receptors (EGFRs), thereby contributing to the repair and regeneration of the myocardium and damaged cardiomyocytes [73]. Furthermore, miR-133a is known to regulate DNA methylation in diabetic hearts [74]. Cationic polyethylenimine (PEI) and anionic miR-133a form self-assembled complexes at an miR:PEI ratio of 1:10. PLGA–PEI nanocarriers co-encapsulating azacitidine and miR-133a (ca. 298 nm in diamter) induced direct epigenetic reprogramming of cardiac fibroblasts into cardiomyocyte-like cells [75]. FITC-labeled PLGA nanocarriers were internalized into human cardiac fibroblasts (HCFs) and human cardiomyocytes (HCMs), as confirmed by confocal microscopy and flow cytometry [75]. Cy5-labeled miR-133a was also successfully internalized into both HCFs and HCMs. However, the precise internalization mechanisms of azacitidine- and miR-133a-loaded PLGA–PEI nanocarriers remain unclear. If internalization occurs via endocytosis, the subsequent endosomal or lysosomal escape mechanisms of miR-133a also remain to be elucidated.

Accordingly, PLGA–PEI nanocarriers co-delivering the DNMT inhibitor azacitidine and cardiogenic miR-133a enable efficient intracellular delivery to cardiac fibroblasts and cardiomyocytes, inducing epigenetic reprogramming toward cardiomyocyte-like cells after myocardial infarction. This nanodelivery system facilitates combined small-molecule and nucleic acid transport, although the precise cellular uptake and endosomal escape mechanisms remain to be clarified.

### 2.7. Promising Strategies for Epigenetic Drug Delivery

**Figure 17:**
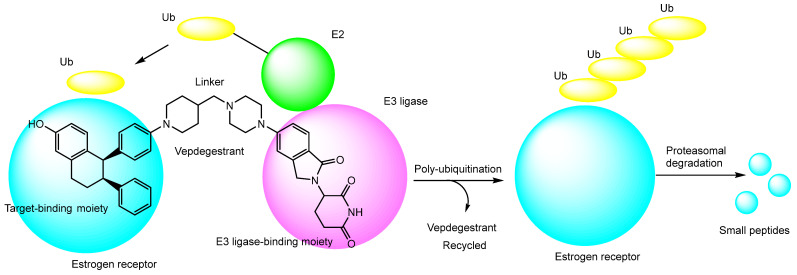
The degradation pathway mediated by proteolysis-targeting chimeras (PROTACs), exemplified by vepdegestrant (ARV-471). Vepdegestrant is composed of a target-binding moiety that recognizes the estrogen receptor (indicated by large light blue circles) and an E3 ligase-binding moiety connected through an appropriate chemical linker. When the estrogen receptor and an E3 ligase (indicated by magenta circles) are brought into close proximity by the PROTAC molecule, the estrogen receptor becomes ubiquitinated with ubiquitin (Ub; indicated by yellow ellipses) via an E2 conjugating enzyme (indicated by green circles). The polyubiquitinated estrogen receptor is subsequently degraded into small peptide fragments (indicated by small light blue circles) by the proteasome. The liberated vepdegestrant molecule can then be recycled to mediate additional rounds of target degradation.

The strategies for selective and non-toxic epigenetic drug delivery rely on a variety of mechanisms, including carrier-mediated transport for conjugates containing transporter-recognition units (Section 2.1) and receptor-mediated endocytosis for conjugates with ligands (Section 2.2), CPPs (Section 2.3),THPs (Section 2.4), monoclonal antibodies (antibody–drug conjugates) (Section 2.5), and nanoparticle-based conjugates (Section 2.6), as described in the preceding sections (Table 1). Recently, proteolysis-targeting chimeras (PROTACs) have emerged as a novel therapeutic modality; consequently, targets that were previously considered undruggable by conventional occupancy-based approaches may now become accessible for drug discovery. PROTAC molecules consist of a target protein-binding ligand and an E3 ubiquitin ligase-binding ligand connected by a suitable chemical linker. Through the ubiquitin–proteasome system, PROTACs promote ubiquitination of the target protein, leading to its selective degradation by proteasomes (Figure 17) [76]. Vepdegestrant (ARV-471) (Figure 17) is an orally active PROTAC-based estrogen receptor degrader that, notably, became the first in the world to successfully complete a phase III clinical trial in 2025, developed by Arvinas [77]. Importantly, epigenetic regulatory proteins—such as HDACs—can also serve as potential proteins of interest (POIs) for PROTAC-mediated degradation, offering new directions for epigenetic drug design.

(xv) Antibody-conjugated anti-HDAC PROTACs represent an innovative class of epigenetic modulators [78]. Enzymatic degradation of target proteins by PROTACs is generally more effective than competitive inhibition by conventional enzyme inhibitors. Peptide-based PROTACs can traverse cellular membranes through the incorporation of CPPs, whereas small-molecule PROTACs can enter cells via passive diffusion [79]. However, such PROTACs typically lack cell-type selectivity in their distribution [79]. Antibody–PROTAC conjugates overcome this limitation by exploiting the EPR effect, which facilitates their passive accumulation in tumor tissues, followed by active cellular uptake via receptor-mediated endocytosis through antibody–receptor interactions. Once internalized, small-molecule PROTACs released from the conjugates can diffuse across endosomal membranes into the cytosol, where they induce HDAC degradation through the ubiquitin–proteasome system.

Therefore, Antibody–PROTAC conjugates enable tumor-selective delivery of anti-HDAC PROTACs by combining EPR-mediated tumor accumulation with antibody-driven receptor-mediated endocytosis. After intracellular release, the PROTACs access the cytosol to induce targeted HDAC degradation via the ubiquitin–proteasome system, improving efficacy and cell-type specificity over conventional PROTACs.

## 3. Conclusions

Epigenetics plays a vital role in biological development, differentiation, and morphology. Genetic processes are regulated by the biological machinery governing transcription and translation, as described in the central dogma of molecular biology and grounded in structural principles [21,22]. In contrast, epigenetic mechanisms modulate this biological machinery without altering the underlying DNA sequence, thereby contributing to phenotypic diversity [9,10]. However, dysregulation of epigenetic control can lead to pathological states, including cancer, metabolic disorders, and neurodegenerative diseases [9,10]. Epigenetic drugs are therefore expected to restore disrupted homeostasis and rebalance cellular equilibrium through the re-establishment of multilayered regulatory networks. Notably, epigenetic modulators have the potential to correct disease states fundamentally, particularly in neurodegenerative disorders such as Alzheimer’s disease (AD) and Parkinson’s disease (PD) [80,81]. Several small-molecule epigenetic modulators, such as HDAC inhibitors and DNMT inhibitors, have been developed. Nevertheless, few have achieved recent clinical approval, whereas siRNAs have emerged as a promising new therapeutic modality. Drug development requires careful consideration of absorption, distribution, metabolism, excretion, and toxicity (ADMET) properties. HDAC and DNMT inhibitors often suffer from poor membrane permeability, low solubility, and off-target effects due to their hydrophilic nature. Conversely, siRNAs and antisense oligonucleotides are enzymatically unstable because of degradation by serum RNases. To overcome these limitations, the introduction of functionalized accessory units has been explored, using delivery vectors such as transporter-recognition motifs, CPPs, THPs, monoclonal antibodies, and nanoparticles. These modifications have demonstrably improved ADMET characteristics, as discussed earlier. Among these strategies, nanoparticle conjugation has shown particularly significant progress, enabling the revival of compounds whose clinical development was previously suspended.

Invasive or toxic treatment approaches, such as surgery, craniotomy, or conventional chemotherapeutics with severe side effects, can compromise patients’ quality of life. In contrast, the philosophy of Taoism emphasizes harmony with natural systems. Likewise, in drug design, it is essential to work synergistically with intrinsic biological mechanisms in a biocompatible and non-invasive manner, rather than overriding them. Medicinal chemists and pharmaceutical scientists are thus encouraged to design innovative epigenetic agents capable of addressing diseases such as AD, PD, and cancer at their root causes through integrated pharmacodynamic and pharmacokinetic strategies.

## Figures and Tables

**Table 1:** Delivery strategies for epigenetic drugs using functionalized accessory unit conjugates. Examples shown are primarily selected from studies reporting favorable delivery outcomes.

#	Formulation	Vector	Internalization Mechanism	Cargo	Status	References
i	A pyrilamine derivative with the benzamide zinc-binding group	Transporter recognition unit	Carrier-mediated transport	HDAC inhibitor	Basic research (in vitro, in situ brain perfusion assay)	[27]
ii	HDAC inhibitor conjugated with pteroic acid through carbon chain	Ligand (pteroic acid)	Receptor-mediated endocytosis	HDAC inhibitor	Basic research (in vitro)	[34]
iii	Hydroxamic acid-modified peptides such as H4(5–19)K12Asuha (Ac-KGGKGLGXGGAKRHR-W-NH2, X=Asuha)	Cell-penetrating peptides	Receptor-mediated endocytosis	HDAC inhibitor (Asuha)	Basic research (in vitro)	[42]
iv	Panobinostat (LBH589) and scriptaid, concomitantly with Delta24-RGD	RGD-motif	Receptor-mediated endocytosis	HDAC inhibitors (panobinost, scriptaid)	Basic research (in vitro)	[44]
v	Antibody–drug conjugates	Antibodies (cetuximab, trastuzumab)	Receptor-mediated endocytosis	HDAC inhibitors (ASAHA, dacinostat)	Basic research (in vitro)	[47]
vi	Antibody–drug conjugates (ctx-NH-4, ctx-NH-7)	Antibody (cetuximab)	Receptor-mediated endocytosis	Thiol-based HDAC inhibitor	Basic research (in vitro)	[50]
vii	TA-loaded chitosan-based nanoparticles (Chi-TA-NPs) (567.0 ± 25.84 nm in diameter)	Unknown	Unknown	Tannic acid (expression of DNA methyltransferase)	Basic research (in vitro)	[54]
viii	SAHA-loaded black phosphorus nanoparticles camouflaging with M1 macrophage membranes (MBS) (ca. 100 nm in diameter)	Integrin α4β1 on M1 macrophage membranes	Receptor-mediated endocytosis	HDAC inhibitor (SAHA)	Basic research (in vitro)	[55]
ix	RGD peptide-modified poly(D,L-lactide-co-glycolide) (PLGA) nanoparticles (ARNPs) loaded with JQ1 (108.39 ± 5.82 nm in diameter)	RGD peptide	Receptor-mediated endocytosis	BET bromodomain inhibitor (JQ1)	Basic research (in vivo)	[56]
x	Self-assembled nanoparticles composed of isoalantolactone with hydroxamic acid	Unknown	Unknown	Signal transducer and activator of transcription 3 (STAT3), HDAC inhibitor	Basic research (in vitro, in vivo)	[57]
xi	PLGA-based spherical nanoparticles encapsulating decitabine covered with anti-PD-L1 antibody and macrophage membrane (Dec@PLGA@aMM) (95.7 ± 9.1 nm in diameter)	Antibody (anti-PD-L1 antibody)	Receptor-mediated endocytosis	DNA methyltransferase inhibitor (decitabine)	Basic research (in vivo)	[61]
xii	Decitabine-encapsulated PLGA core nanoparticles hybridized with TET1 gene-encoding plasmid DNA (ca. 200 nm in diameter)	Unknown	Unknown	DNA methyltransferase inhibitor (decitabine), TET1 gene-encoding plasmid DNA	Basic research (in vitro)	[64]
xiii	siHSP47 and siHMGB1-encapculated ionizable polymeric micelles modified with a fibroblast activation protein inhibitor (FAPi-IPMs) (less than ca. 175 nm in diameter)	Fibroblast activation protein inhibitor	Receptor-mediated endocytosis	siRNAs (reduction of collagen secretion and liver inflammation)	Basic research (in vitro)	[71]
xiv	Azacitidine and miR-133a-encapsulated PLGA-PEI nanocarriers (ca. 298 nm in diameter)	Unknown	Unknown	DNA methyltransferase inhibitor (decitabine), miRNA (DNA methylation regulation)	Basic research (in vitro)	[75]
xv	PROTAC–antibody conjugates	Antibody	Receptor-mediated endocytosis	HDAC degrader	Under analysis in Tashima lab	-

## Data Availability

Data available in a publicly accessible repository. The data presented in this study are openly available in References below. ClinicalTrials.gov identifier can be found at https://clinicaltrials.gov/.
